# Mobile phone handover data for measuring and analysing human population mobility in Western Ethiopia: implication for malaria disease epidemiology and elimination efforts

**DOI:** 10.1186/s12936-022-04337-w

**Published:** 2022-11-11

**Authors:** Werissaw Haileselassie, Ashagrie Getnet, Hiwot Solomon, Wakgari Deressa, Guiyun Yan, Daniel M. Parker

**Affiliations:** 1 School of Public Health, Addis Ababa University, Addis Ababa, Ethiopia; 2 Institute of Technology, Addis Ababa University, Addis Ababa, Ethiopia; 3 Ministry of Health, Addis Ababa, Ethiopia; 4 Program in Public Health, College of Health Sciences, University of California at Irvine, Irvine, CA 92697 USA

**Keywords:** Human mobility, Handover, Malaria, Cellular networks, Western Ethiopia

## Abstract

**Background:**

Human mobility behaviour modelling plays an essential role in the understanding and control of the spread of contagious diseases by limiting the contact among individuals, predicting the spatio-temporal evolution of an epidemic and inferring migration patterns. It informs programmatic and policy decisions for effective and efficient intervention. The objective of this research is to assess the human mobility pattern and analyse its implication for malaria disease epidemiology.

**Methods:**

In this study, human mobility patterns in Benishangul-Gumuz and Gambella regions in Western Ethiopia were explored based on a cellular network mobility parameter (e.g., handover rate) via real world data. Anonymized data were retrieved for mobile active users with mobility related information. The data came from anonymous traffic records collected from all the study areas. For each cell, the necessary mobility parameter data per hour, week and month were collected. A scale factor was computed to change the mobility parameter value to the human mobility pattern. Finally, the relative human mobility probability for each scenario was estimated. MapInfo and Matlab softwares were used for visualization and analysis purposes. Hourly travel patterns in the study settings were compared with hourly malaria mosquito vector feeding behaviour.

**Results:**

Heterogeneous human movement patterns were observed in the two regions with some areas showing typically high human mobility. Furthermore, the number of people entering into the two study regions was high during the highest malaria transmission season. Two peaks of hourly human movement, 8:00 to 9:00 and 16:00 to 18:00, emerged in Benishangul-Gumuz region while 8:00 to 10:00 and 16:00 to 18:00 were the peak hourly human mobility time periods in Gambella region. The high human movement in the night especially before midnight in the two regions may increase the risk of getting mosquito bite particularly by early biters depending on malaria linked human behaviour of the population.

**Conclusions:**

High human mobility was observed both within and outside the two regions. The population influx and efflux in these two regions is considerably high. This may specifically challenge the transition from malaria control to elimination. The daily mobility pattern is worth considering in the context of malaria transmission. In line with this malaria related behavioural patterns of humans need to be properly addressed.

**Supplementary Information:**

The online version contains supplementary material available at 10.1186/s12936-022-04337-w.

## Background

Human movement and travel (HMT) patterns are important for a wide variety of fields [1]. For example, an understanding of travel patterns can be useful for regional planning and economics. Commuting patterns to and from work, can be important with regard to civil engineering [2, 3]. Travel into and from regions with elevated risk of exposure to contagious pathogens or harmful environmental contaminants is an important topic for epidemiological studies and for planning public health interventions [4–6]. Changing patterns of human mobility can affect disease transmission dynamics for illnesses that are driven by human aggregation.

Mobile phone data can capture epidemiologically relevant data, including temporal and spatial dynamics of human mobility. It is a unique data source that can be used to characterize the temporal clustering effect of disease and importation risk mapping through understanding travel behaviour and population flux over time. Mobile phone data have been used to infer that movement patterns predict seasonal variations of the occurrence of infectious diseases [7]. Human movement between areas of heterogeneous transmission intensity has an important role in the spatial and temporal dissemination of diseases, including those that are vector born like malaria. It results in parasite importation which may lead to elevated malaria transmission, renewal or preservation of malaria transmission including spread of drug resistance [8]. Moreover, human movement behaviour on local scale may facilitate residual transmission depending on vector bionomics and transmission intensity. This can be an impediment for national malaria control programmes striving for elimination [8–10].

While HMT is important for a wide variety of disciplines, empirical travel data are difficult to collect and are subject to a variety of biases. Many studies qualitatively explored the impact of human travel on health, economics and development in Africa though there is paucity of quantitative information on human mobility in this continent [8]. Historically, exploring HMT in detail has been challenging because data collection methods consisted of cumbersome manual travel surveys or space-time diaries [11]. In recent years, there has been an increase in studies incorporating empirical measurements through the use of wearable GPS (global positioning system) loggers or from mobile phone data [7, 12–14]. Eagle et al. used data from cell phones to study individual mobility and concluded that human trajectories are predictable in a sense that time and space are occasionally repeated, like going from home to work, restaurants, shopping malls or gas stations, and occasional vacations [15]. Gonzalez et al. investigated human movements based on a sample of 100,000 randomly selected individuals, covering a six-month time period using mobile phone data. They found that human mobility patterns show a high degree of spatial and temporal regularity. Moreover, individuals typically return to a few highly frequent locations and most travel trajectories are rather short in terms of distance and travel time [16].

Mobile phone networks are dynamic networks that are created by connections between users’ mobile devices. Use of mobile phone data for measuring human travel and movement patterns is facilitated by the widespread ownership and use of mobile phones in much of the world. The opportunistic information exchanges between mobile users are highly relevant to their mobility patterns [17]. Use of mobile phone data is increasingly generalizable as mobile phone ownership is increasing globally. These features help and has triggered a range of studies: to analyse human mobility pattern and spatial interactions in both physical space and time [18, 19].

User mobility and handover are two important functions in mobile networks that provide seamless connectivity for the mobile device when moving from one cell phone tower to another. Hence, mobile phone handovers and associated networks were used for a quantitatively grounded conception of human mobility in the two places of heightened importance with regard to malaria epidemiology in Ethiopia: Benishangul-Gumuz and Gambella Regions. A thorough investigation of human travel patterns and population flux over time is provided from the live Global System for Mobile Communications (GSM) and Universal Mobile Telecommunication System (UMTS) networks. Furthermore, we also compared hourly travel patterns in these two locations with hourly malaria mosquito vector feeding behaviour, with implications for the ecology of malaria in these two settings (which have the highest burdens of malaria in Ethiopia). This study is the first of its kind in Ethiopia, laying the foundation for further research on the area by providing baseline information and with considerations for malaria public health efforts.

## Methods

### Study setting

**Fig. 1:**
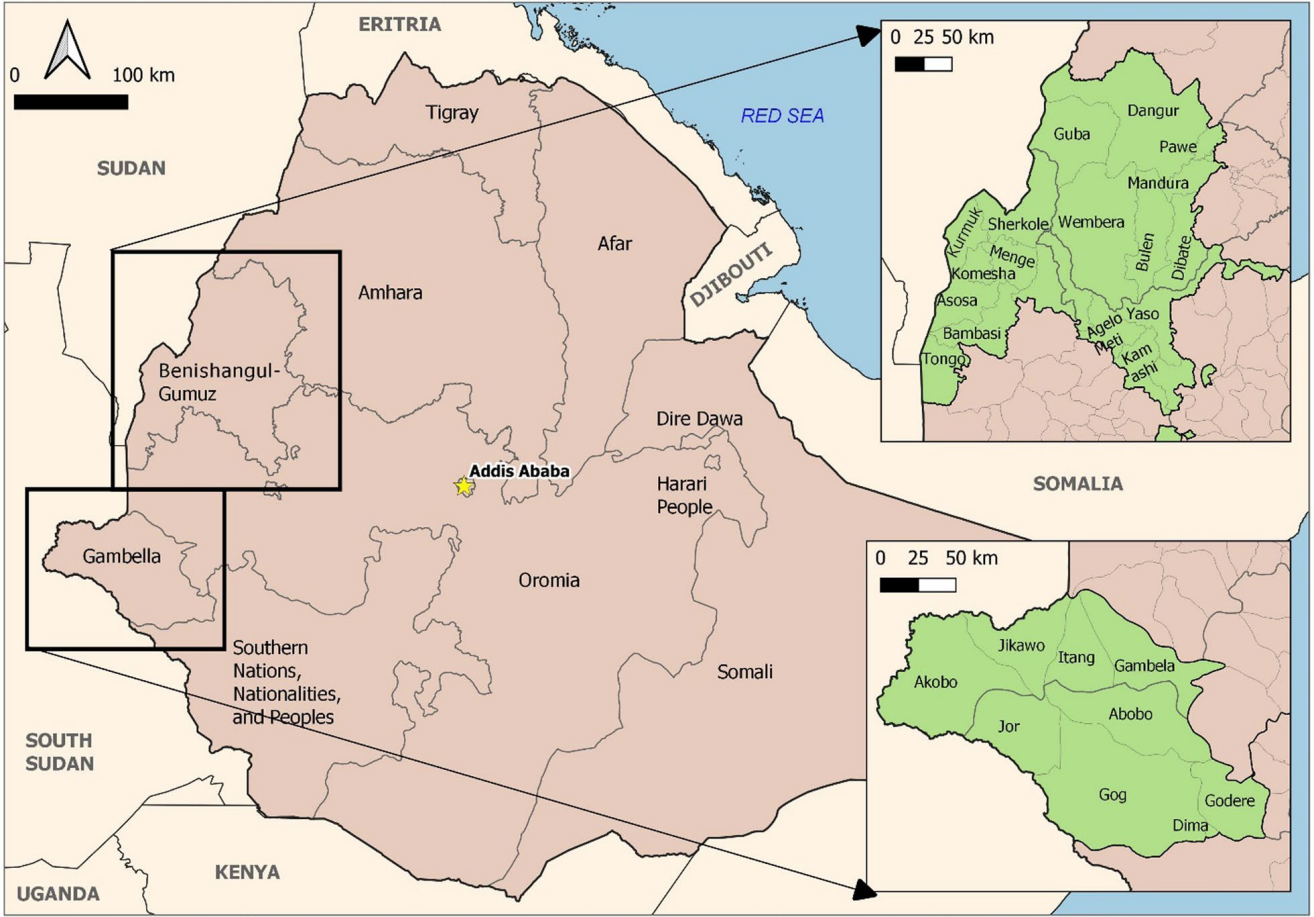
Study locations and major administrative units

This study was conducted in Benishangul-Gumuz and Gambella regions of Western Ethiopia (Fig. 1). These two regions were selected because they have the heaviest malaria burdens in Ethiopia and are regionally important with regard to malaria epidemiology. Around half the population of Ethiopia, 52.9 of every 100 inhabitants, subscribed to Ethio Telecom’s mobile service. The total population of Benishangul-Gumuz is estimated to be 1,109,565 of which 50.8% are males and 78.4% of the population lives in rural areas. There are 10 urban areas in the region. Assosa is the capital of the region which is located at a distance of 665 km from Addis Ababa, Ethiopia. The region consists of 20 districts and three city administrations in 3 zones. Pawe, Metekel, Assosa and Kamashi are major population centres (towns) in the region. The total area of the region is about 49,289.46 km^2^ with greater than 784,345 inhabitants and an estimated population density of 15.91 people per km2. Its elevation ranges from 580 to 2731 m. The hottest period is from February to April with a range of 35 ℃-40 ℃ The annual rainfall ranges from 800-2000 mm. Approximately 97% of the population is at risk of getting malaria while 98% of all the Kebeles in the region are malarious. Currently, various public and private development activities such as agriculture and mining are being undertaken [20].

Gambella region has 3 zones with 13 districts and one city administration: Gambella, Abobo, Lare, Itang, Jor, Akobo towns. Gambella has an area of 29,782.82 km^2^ with 13 districts and one town administration. There are 25 urban and 237 rural areas in the region. The total population of the region is 468,017. Moreover, there are about 271,000 refugees in the region [21]. The mean annual temperature varies from 17.3 ℃–28.3 ℃. The annual rainfall of the region ranges from 900-1500 mm and 1900-2100 mm at lower and higher altitudes, respectively. The elevation ranges from a low of 300 m to a high of 2200 m. There is perennial transmission of malaria in all Kebeles of the region. Agricultural and mining development activities are ongoing in the region [22].

### Data acquisition

This study was conducted based on an anonymized dataset of nearly 121 and 17 cellular towers (each with geographical coordinates) in Benishangul-Gumuz and Gambella regions, respectively. Both inter-cell and intra-cell handover data were obtained from the sites. In areas where there is low traffic distribution and sparsely distributed towers (i.e. rural areas) only intra-cell hand off were used. A total of 330,732,648 handovers were observed from Benishangul-Gumuz region while 696,010,712 handovers were observed in Gambella region. These data come from Ethiotelecom, the sole mobile phone provider in Ethiopia.

Their distribution varies based on the spatial population density distribution, with more clusterin in urban areas and less clustering in suburban and rural areas of the two regions. By using the OSS network data, it was possible to retrieve mobile active users with mobility related information (HO, active user distribution per cell, traffic distribution, TCH) from February in 2019 to late August of that year for Benishangul-Gumuz region (7 months total) and January to October of 2020 (10 months total) for Gambella region. Our goal was to capture both short (hourly) and long-term trends or seasonality in movement behaviours, and the study period includes all important public or religious holidays that may have different mobility patterns.

To formulate users mobility, the most important data are the handover (HO), number of active user in the specified time period, the traffic distribution all over the network; and Location Update (LU) statistics. While the former reflect the movement of connected users and LU do the same only for idle users (note that connected users do not use the SDCCH to update their location, but the TCH). Moreover, a mobility model contains a set of rules that permit one to predict statistically how long a call will hold a channel in a cell and if/when this call will originate a handover generation request towards the nearest cell.

The dataset is based on anonymous traffic records collected from all of the study area. Each record includes a timestamp, anonymous cell ID, and location area code (LAC). The abundant information in daily handover records amounts and provided a sufficient dataset foundation for analysis and evaluation. Furthermore, the dataset is sorted and classified by temporal and spatial properties, like daytime/night and rural/urban/dense urban area. Similarly, the rural areas considered the places like country sides and villages while the urban areas merely include cities.

Data on host-seeking behaviours of mosquitoes were obtained from the research teams’ entomological research work as well as from other published literature [23, 24]. Briefly, mosquito collection was conducted for a total of eight man-nights with the collection time extending from 18:00 to 06:00 h in Gambella. A similar study was conducted by another team in Benishangul-Gumuz. Mosquitoes were collected between July and December 2017 and the collection was conducted from 18:00–6:00.

### Data analysis

In this research, a human mobility algorithm was used to analyse mobile phone level information. Spatial patterns and characteristics of human mobility were investigated at the different spatial and temporal scales. A scale factor was computed to change the mobility parameter value to the human mobility pattern. Human mobility patterns and relative probability of human mobility was determined for different scenarios using parameter values based on plausible assumptions (detailed in Additional file 1) [25–31]. Hourly travel patterns in the study settings was compared with hourly malaria mosquito vector feeding behaviour to understand its implication for ecology of malaria.

### Software

MapInfo Pro 16, Matlab 2018b, and Quantum GIS softwares were used for visualization and analysis purposes. MapInfo was used to process images [to create raster maps] by taking the actual human mobility into consideration. This software can render rasters and has the ability to combine and display, on a single map, data from a variety of sources and make smooth map. To generate a smooth map, the raster used a kernel density estimation method so as to estimate the density of mobility in a given area. This technique estimated the proximity or density of mobility data in a given area.

## Results

### Mean monthly human mobility patterns in Benishangul-Gumuz

**Fig. 2:**
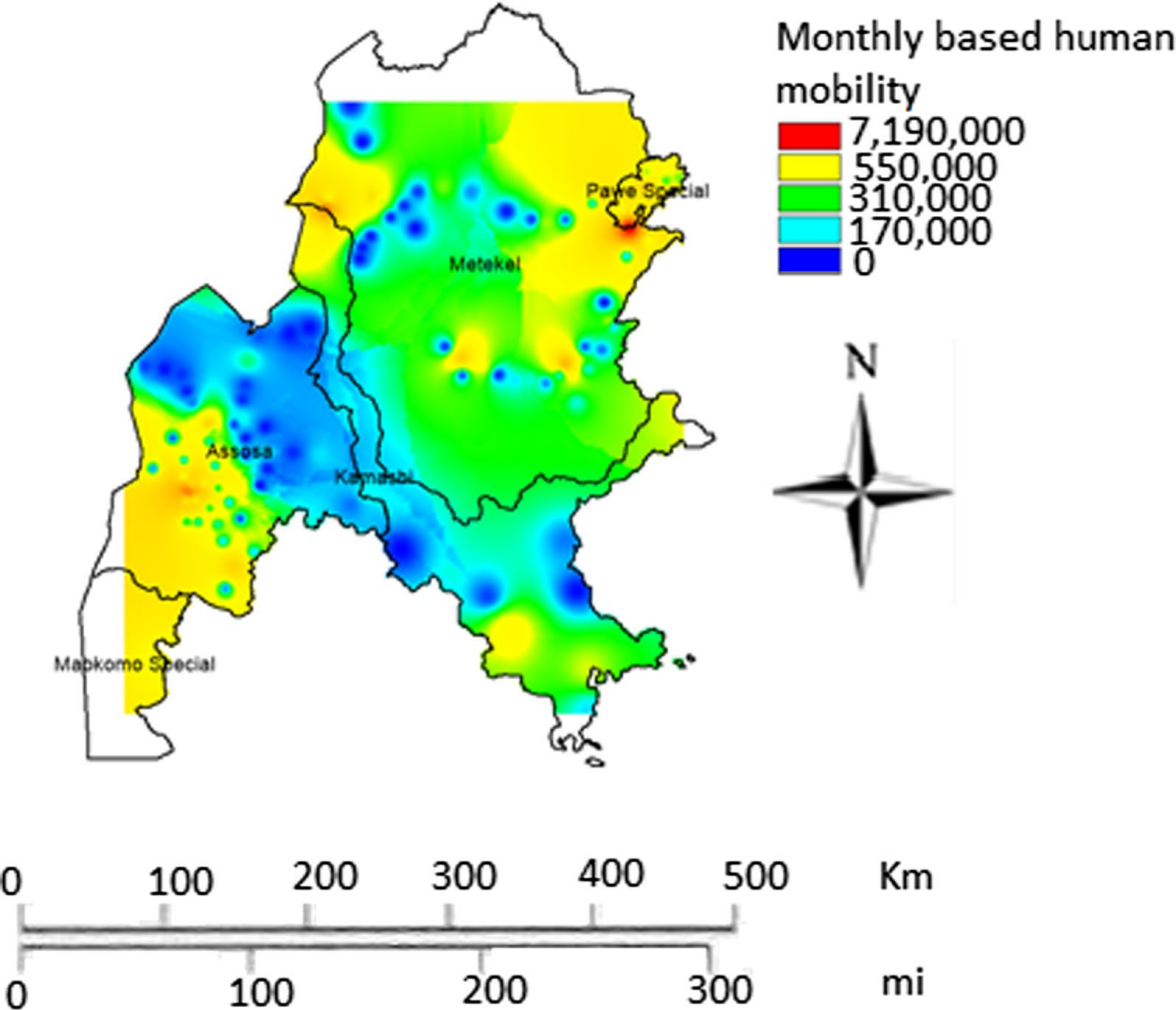
Monthly geographic human mobility pattern in Benishangul-Gumuz region, Ethiopia

The results of our analysis showed that there was heterogeneous human mobility across the different towns in Benishangul-Gumuz region (Fig. 2). High number of human displacement was observed in Assossa and Pawe towns. However, the probability of a jump steadily increased in rural areas and the shapes of the distributions vary from city to city, suggesting either that human movements do not exhibit universal patterns across cities or that distance is not the appropriate variable to model them (< 300 m). 124 trip occurrence per individual per month was observed in the region.

### Mean monthly human mobility patterns in Gambella region

**Fig. 3:**
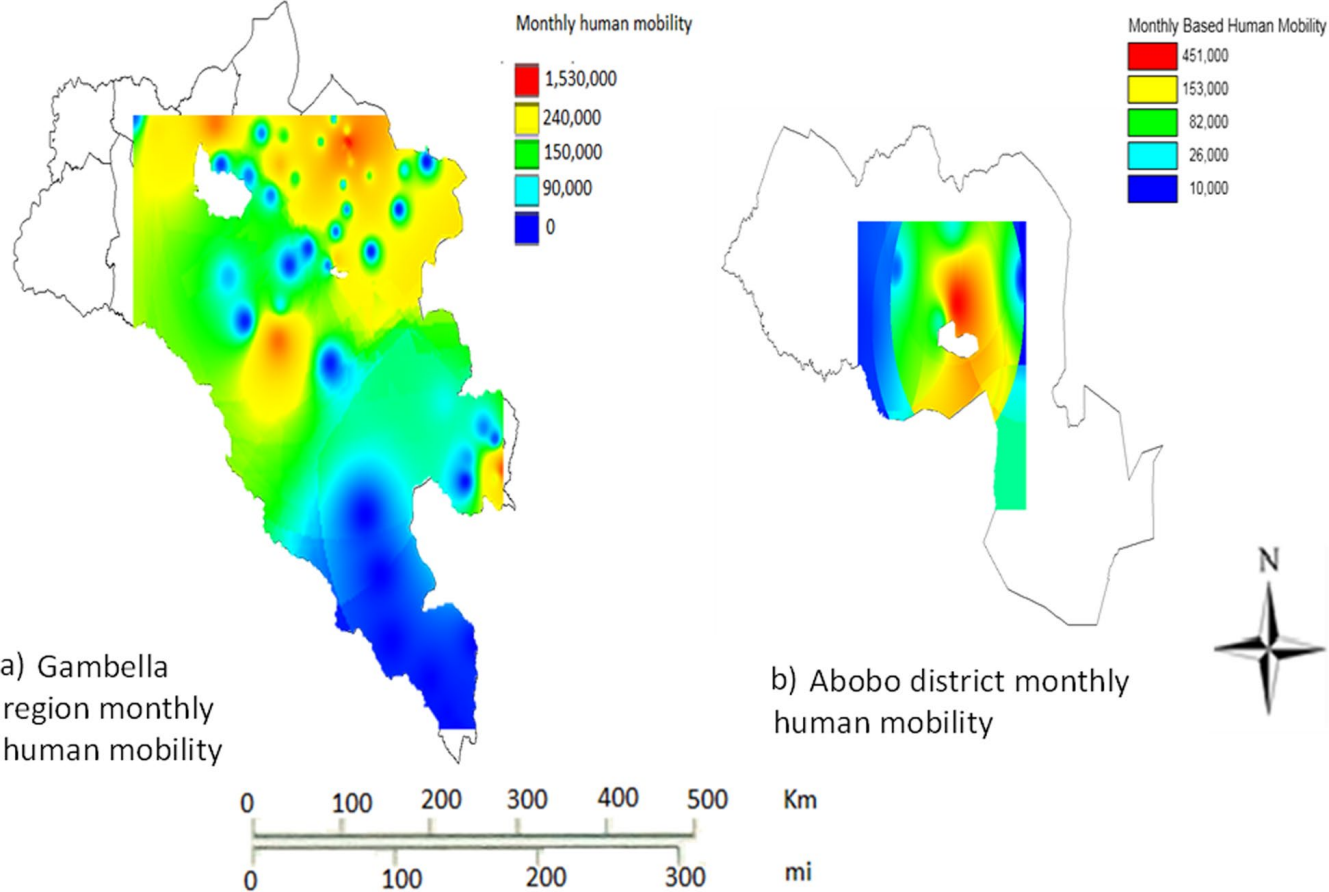
Monthly geographic human mobility pattern in Gambella region (**a**) and Abobo district (**b**) within Gambella region, Ethiopia

Generally, there was high human mobility in Gambella during the study period (Fig. 3a). Great variation in human mobility was observed across the different parts of Gambella region Specifically, high human movement was observed in North, East and Central parts of the region. Accordingly, in Nuer Zone particularly in lare and Itang districts high mobility was observed. These two districts are located at the border between South Sudan and Ethiopia (potentially indicating high cross-border movement). Mobility was also high in Gambella zuriya district where Gambella town, the capital of the region, is found. Similarly, there was considerable human mobility in Abobo district as shown in Fig. 3b). Notably, great variation in human mobility was also observed across the different parts of this district. Moreover, the human mobility was concentrated in the central part of the district where the main town (Abobo town) is located. The human mobility in the district was high around this town. In Abobo district there are large-scale private agricultural farms and infrastructure projects [32]. The number of trip occurrence per individual per month for Gambella region was 139.

### Mean weekly human mobility probability in Benishangul-Gumuz region

**Fig. 4:**
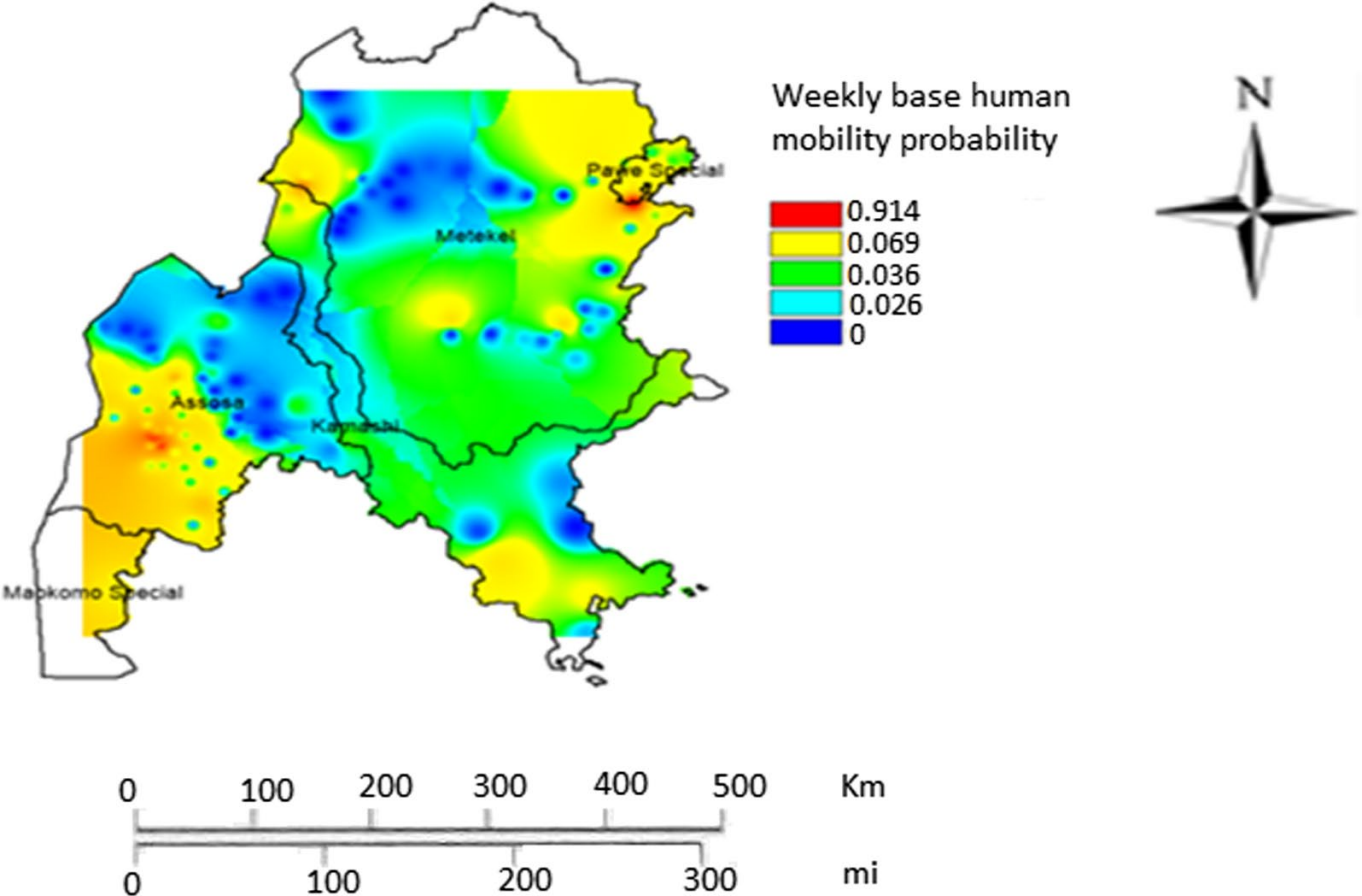
Weekly geographic human mobility probability of Benishangul-Gumuz region

The weekly probability of human mobility was higher in Pawe and Assosa. Moderate mobility probability was observed for most of the central parts of the region while low mobility probability was pronounced around the Western peripheries of the region. In Assosa zone, the low and high human mobility probability extremes were prevailed. In contrast, in the largest zone of the region (Metekel zone) there was primarily moderate human mobility probability. Notably, in the Western fringe where the region shares boarder with Republic of Sudan there are areas with high human mobility (Fig. 4). Mixed colours in some places indicate a mixture of mobility flow pattern of different categories.

### Mean weekly human mobility probability in Gambella region

**Fig. 5:**
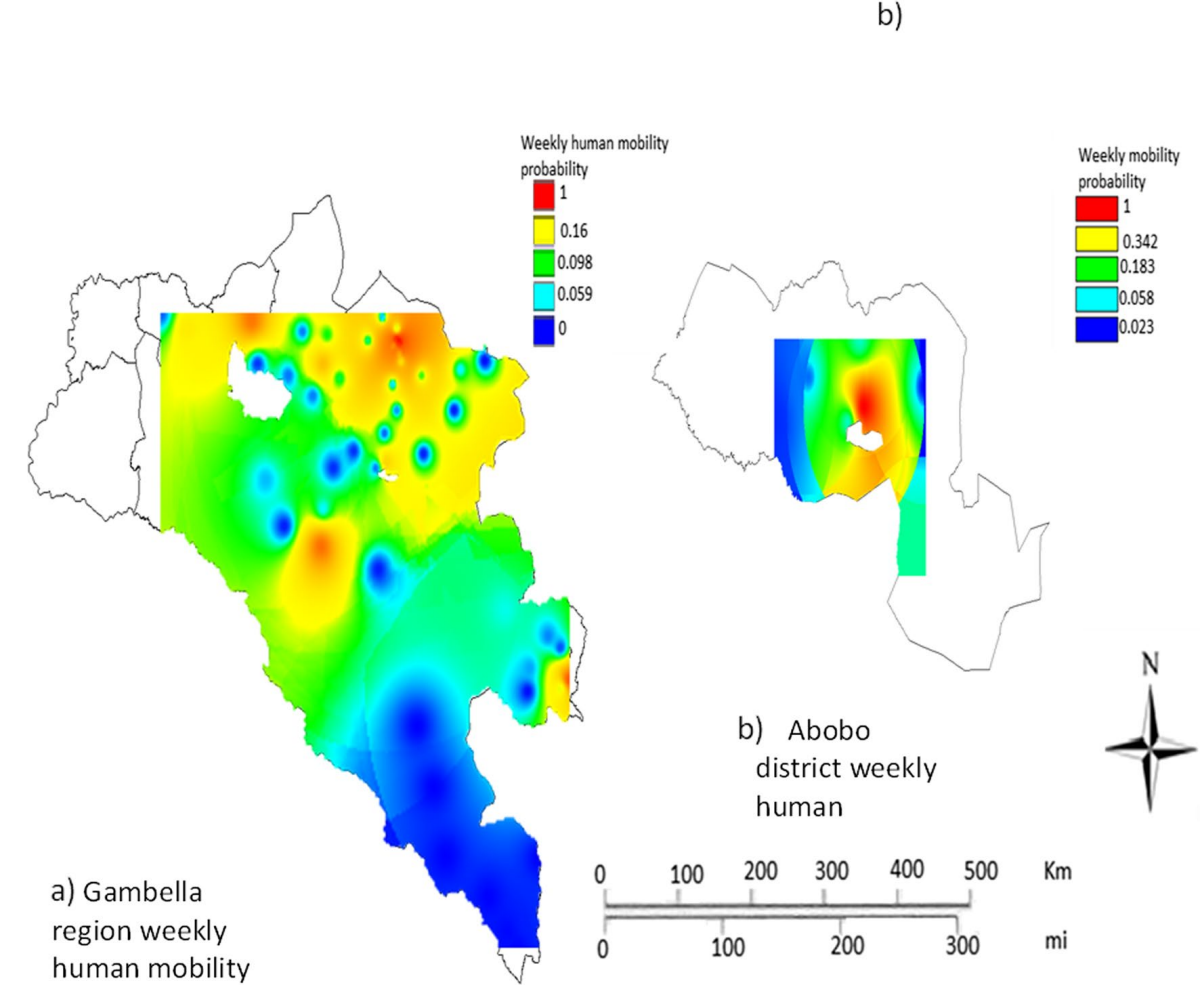
Weekly geographic human mobility for Gambella region** a** and Abobo district** b**, Ethiopia

In Gambella region a high weekly probability of human mobility was observed in the North East and central parts of the region (Fig. 5). Specifically, higher human mobility probability was witnessed in Gambella Zuria, Lare, Gog and Godare districts. Gambella Zuria district including Gambella city showed comparatively higher human mobility probability. The human mobility probability in some parts of Itang and Jikawo districts was notably high. The lowest human mobility was pronounced in Dima district except areas of the district where it borders with Gog district. Moderate to high human mobility was prevailed in Abobo district. Human mobility was highly concentrated in the Central part of the district. High mobility probability was recorded around Abobo town of Abobo district.

### Hourly human mobility patterns in Benishangul-Gumuz region

**Fig. 6:**
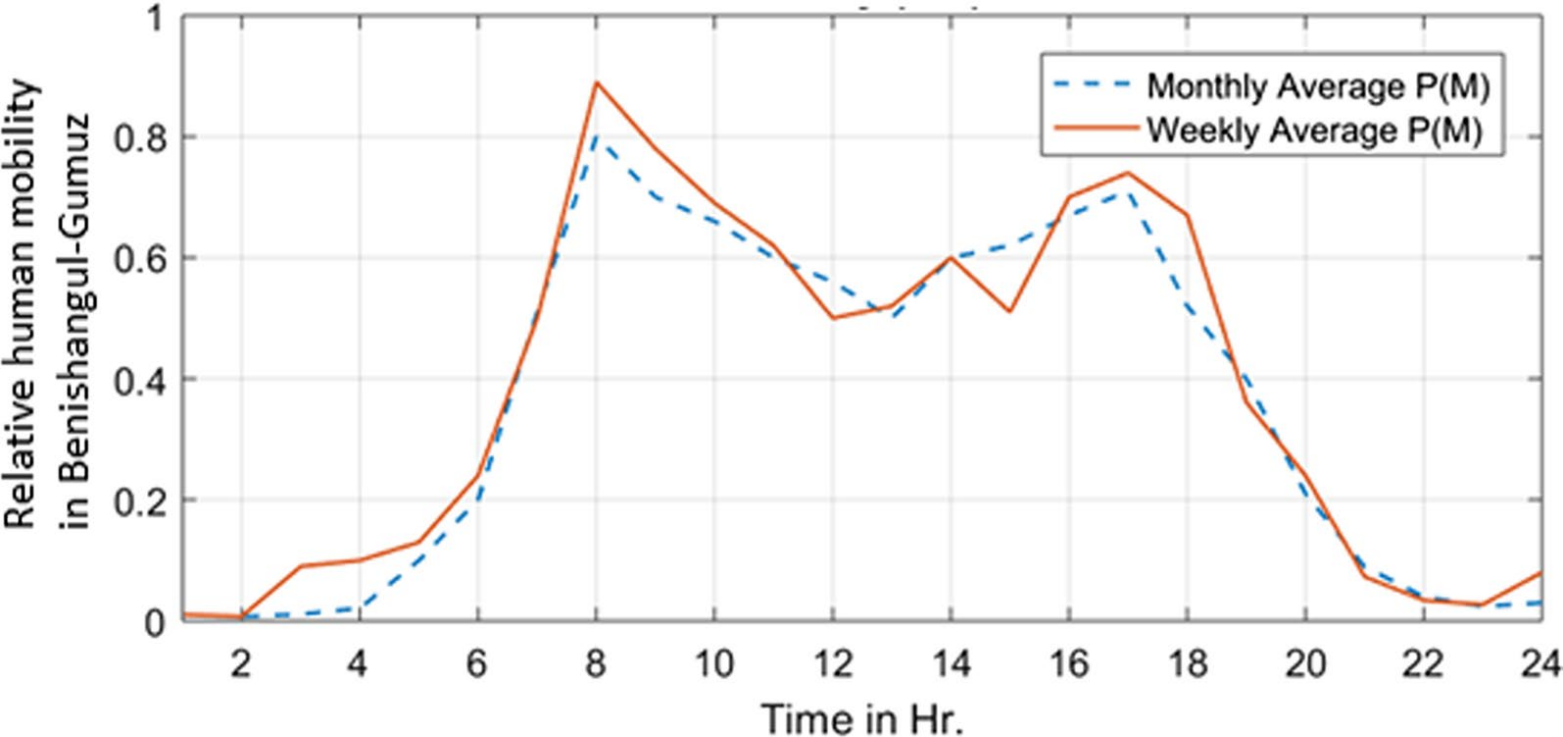
Hourly human mobility probability in Benishangul-Gumuz region, Ethiopia

Figure 6 indicates the 24 h human mobility pattern (fluctuation of human mobility during the day) based on weekly and monthly average for Benishangul-Gumuz region. The solid red line is the average of the 7 weekdays (Monday till Sunday) and the blue dotted line averages the months. Much strong regularity can be observed from the graph. There is very little mobility probability from 20:00 to 6:00. Furthermore, it was observed that two peaks were emerged at different time period within 24 h. The first peak for both scenarios appeared in the period from 8:00 to 9:00. The second peak happened from 16:00 to 18:00 at which most of residents are likely to go back to home. After that, the relative mobility probability decreases, as there are less and less human mobility recorded.

### Hourly human mobility patterns in Gambella region

**Fig. 7:**
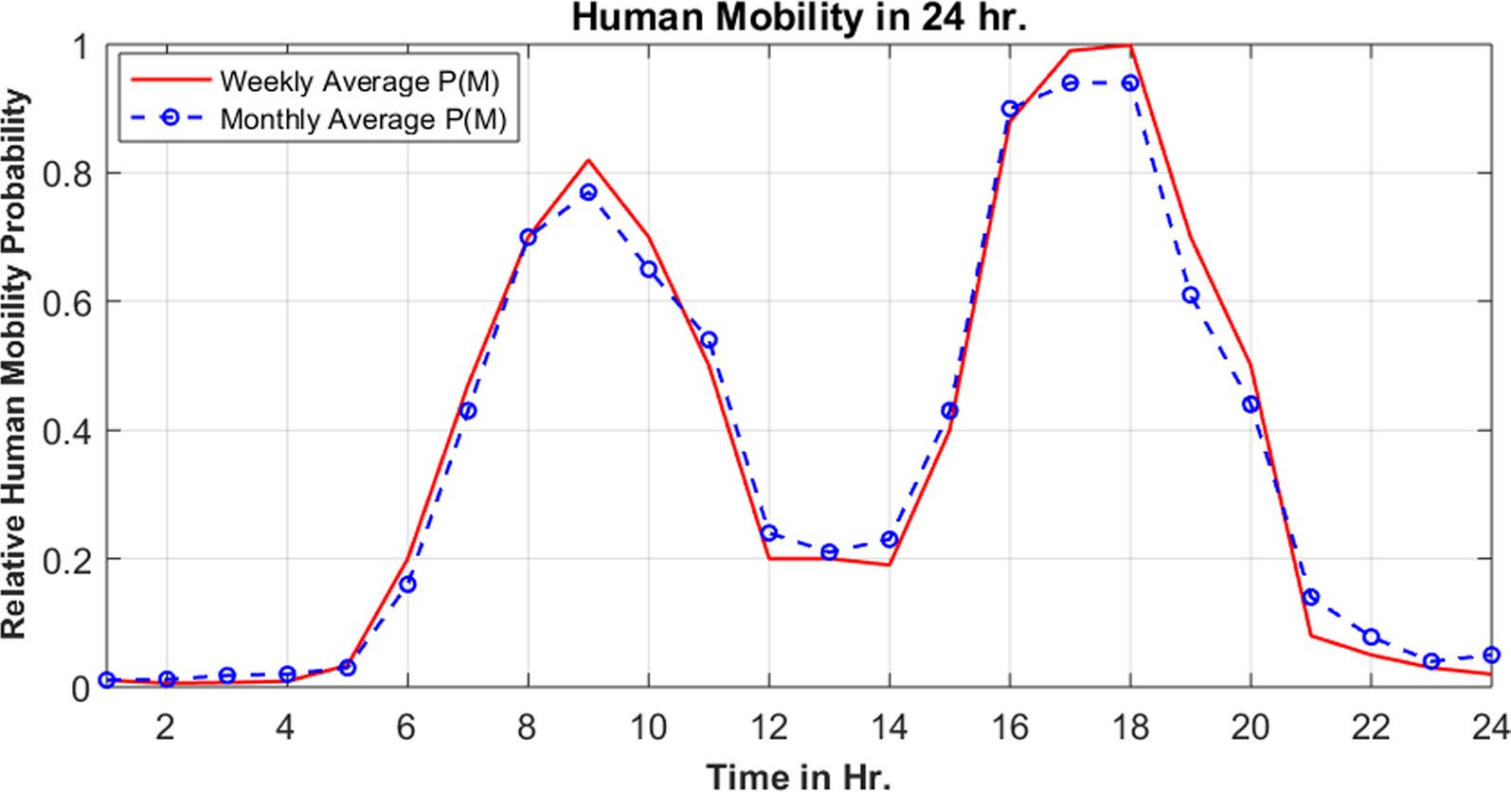
Hourly human mobility probability in Gambella region, Ethiopia

Quite different human mobility probability patterns were observed in Gambella Region (Fig. 7). Unlike Benishangul-Gumuz region, mid-day there was small probability of movement. People tended to spend this time being stationed in one place. Low human mobility probability was observed from 21:00 to 6:00. Two peaks of human mobility probability were evident between 8:00 to 10:00 as well as 16:00 to 18:00 (Fig. 7). Abobo district portrayed similar pattern of mobility probability.

### In-and out-human movement in Benishangul-Gumuz and Gambella regions

**Fig. 8:**
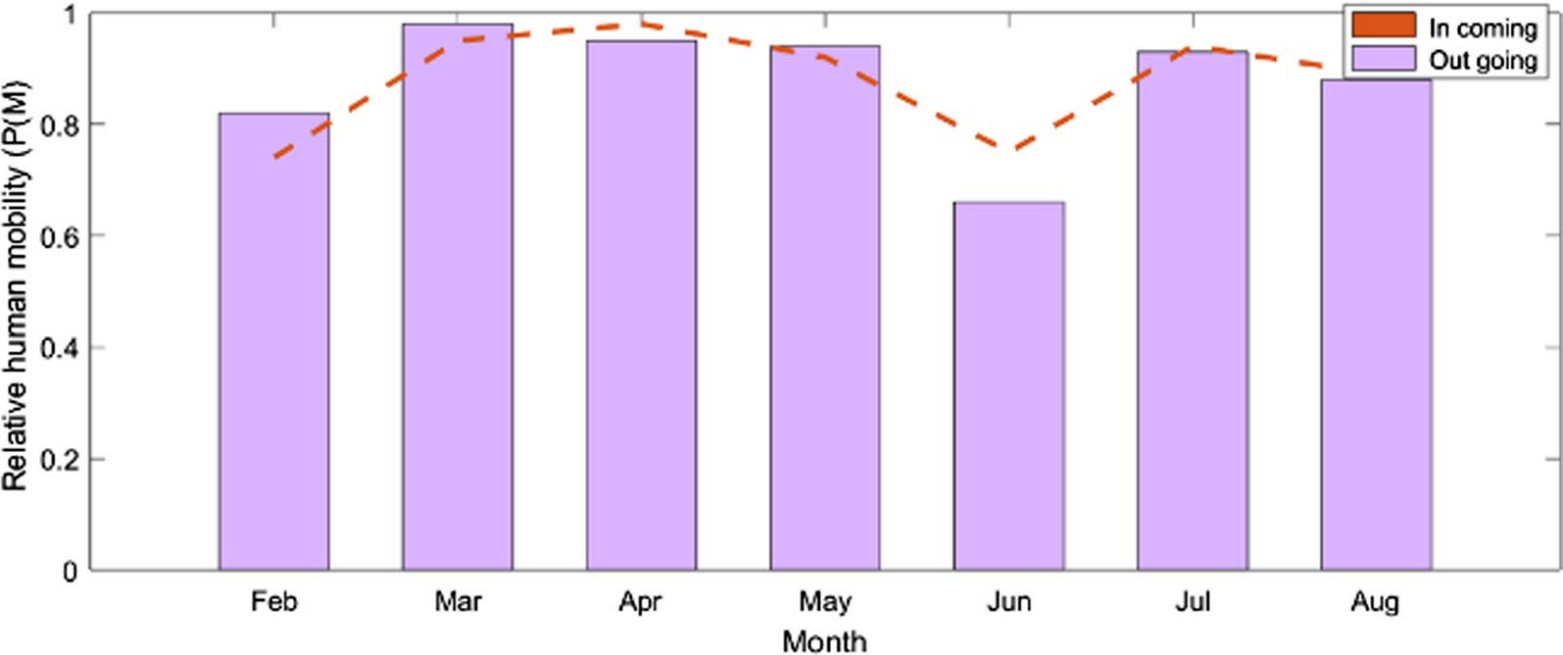
The in- and out-movements in Benishangul-Gumuz region, Ethiopia

**Fig. 9:**
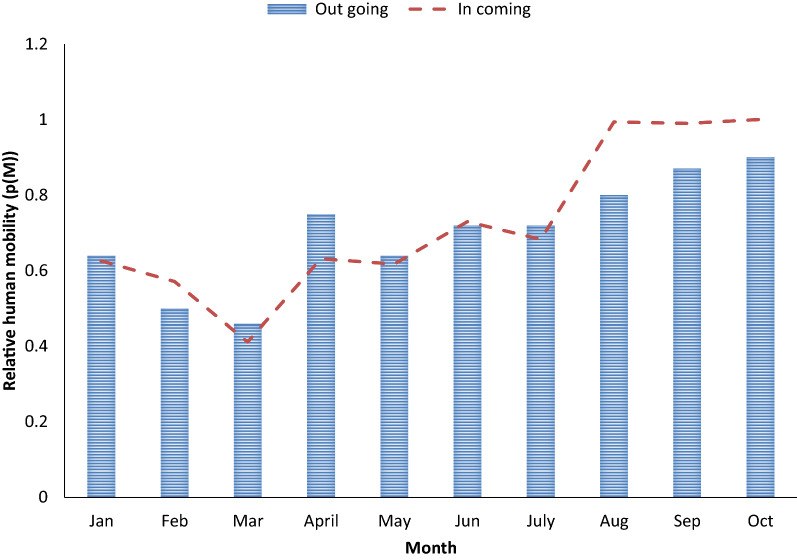
The in and out relative human mobility, Gambella region, Ethiopia

In this case, the same mobile network setup as before was used, but the data of the mobility parameters differs. Figure 8 shows the in and out human mobility in Benishangul-Gumuz region while Fig. 9 depicts the in and out mobility in Gambella region. Human movement probability into the Benishangul-Gumuz region was a little higher than the human mobility probability out of the region. More people enter into the region than those leaving the region. A comparison between the human mobility pattern of rural and urban areas with respect to the number of relative mobility as a function of seven months’ time period was made. Accordingly, high human mobility was observed in urban areas compared to rural area. The mobility probability in March, April and May is almost constant and the lowest was in June. This indicates that the lowest mobility was observed during the rainy season. In Gambella region the number of people entering into the region is high in the months of August, September and October. This period largely falls into a malaria transmission season in the area.

### The malaria situation in Benishangul-Gumuz and Gambella region

**Fig. 10:**
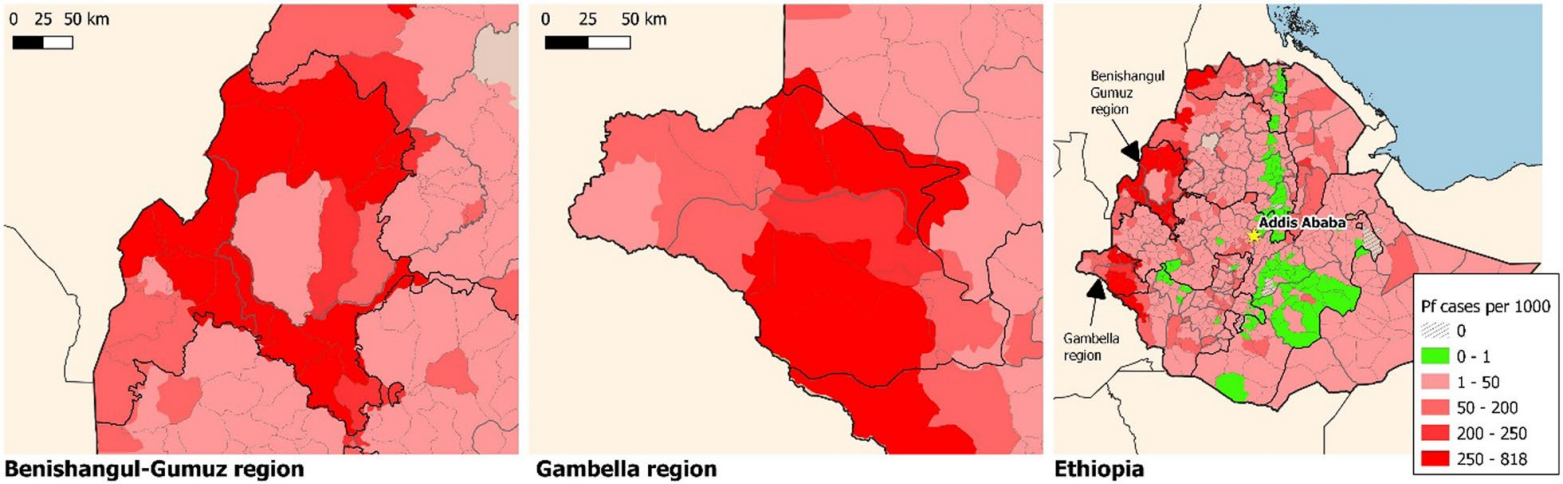
Distribution of reported *Plasmodium falciparum* cases (from 2017) in Benishangul-Gumuz and Gambella regions, Ethiopia

According to the 2019 regional health bureau malaria programme performance report, 97% of the total population of Benishangul-Gumuz region is at risk of malaria [20]. Moreover, every district in the region is malarious (Fig. 10). Similarly in Gambella 12 out of the 13 districts in the region are reported to be malarious [22] (Fig. 10).

### Anopheles mosquito behaviour in the study areas

**Fig. 11:**
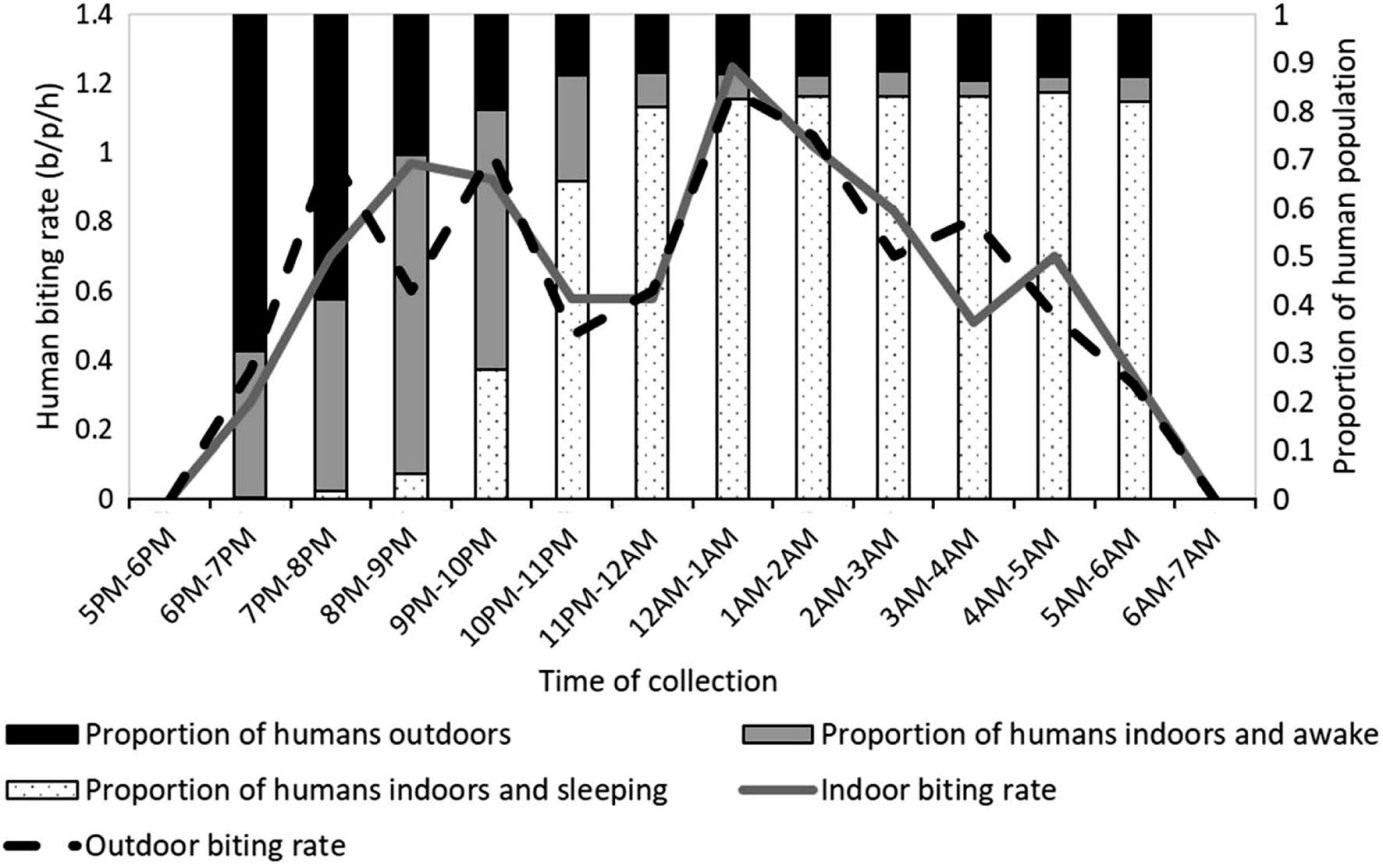
Indoor and outdoor hourly biting rate along with mean proportion of humans outdoors, indoors and awake, and indoors and asleep each hour of the evening in Dangur district of Benishangul-Gumuz, Ethiopia. (Source: Dugassa et al. [23]

A study conducted by Dugassa et al. in Dangur district of Benshangul Region, Ethiopia identified 7 malaria vectors: *Anopheles gambiae *sensu lato (*s.l*.)*, Anopheles pharoensis*, *Anopheles coustani*, *Anopheles demeilloni*, *Anopheles squamosus*, *Anopheles pretoriensis*, *Anopheles natalensis* and *Anopheles christyi*. *Anopheles gambiae s.l.* was identified to be *Anopheles arabiensis* using molecular technique. The study reported that *An. arabiensis,* the major malaria vector*,* bit throughout the night with peak biting periods of 21:00–22:00 and 0:00–2:00. Similar average number of mosquitoes was collected indoor and outdoor during the six months of mosquito collection. The proportion of the population who were outdoors peaked from 6 to 7 pm (Fig. 11). However, the outdoor biting rate was highest at the time of 12 am to 1am. While the proportion of humans indoor and sleeping reached its peak at 4am to 5am, the proportion of people who were indoors and awake attained its highest value from 8 to 9 pm [23].

**Fig. 12:**
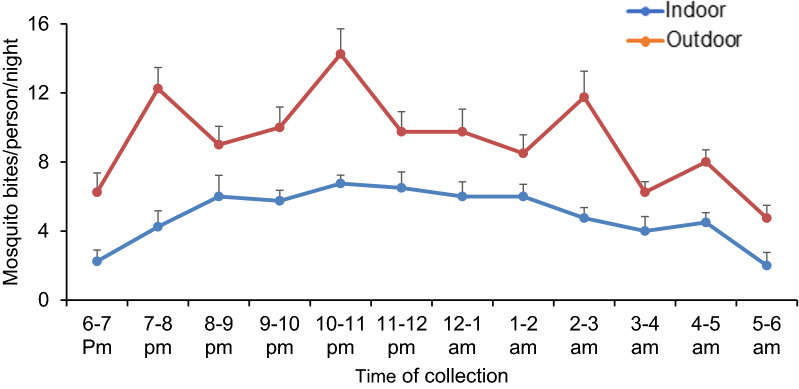
Biting activity of *Anopheles* in Abobo district of Gambella region, Ethiopia. (Source: Haileselassie et al.[24]

In Gambella, the major malaria vectors identified by entomological studies include *An. gambiae s.l., An. pharoensis*, *Anopheles coustani*, *Anopheles nili and Anopheles funestus* [24, 33]. Vector bionomics study in the region indicated that *Anopheles* biting occur throughout the night with a higher outdoor biting rate at each hour of the evening. The peak biting activity was between 9 pm-12 pm (Fig. 12). Based on the human mobility pattern shown above, there is a high human mobility during this peak biting period posing particular risk of mosquito bite [24].

## Discussion

The overarching goal of this study was to empirically quantify human mobility, with regard to its importance for malaria disease epidemiology. For this, geo-referenced temporal mobile phone data were used to assess human movement patterns geographically and temporally (hourly and monthly movements). The results of this study show that cellular network data can provide valuable information on human movement trajectories. Given the current widespread coverage of the use of mobile phone in Ethiopia, and that Ethiotelecom is the sole mobile phone network company in Ethiopia, this data source can serve as a promising source for studies on human mobility, which is important for a wide variety of study areas including infectious diseases.

These results show that human movement patterns in the two study regions differ markedly. There were high amounts of human mobility along the international borders, which may be related to international border crossings, potentially a result of continued hostilities in the Republic of Sudan and South Sudan. The rapidly changing regional geopolitics makes some human population movements relatively unpredictable. Until recently, there was unrestricted free movement of humans along the international border lines. Residents with social ties on both sides of the borders frequently cross those international borders. The overall high weekly and monthly human mobility in the two regions could be attributed to the change in the socio-economic and political environment. The nationwide expansion in road infrastructure over the past few decades has improved transport connections both within and outside of the two regions facilitating human mobility [34, 35]. Given that there are major differences in health care systems and malaria transmission intensities between Ethiopia, the Republic of Sudan, and South Sudan, this could frustrate malaria elimination efforts in Ethiopia (and elsewhere).

Within region variations in human mobility was also highly pronounced. Some of these movements are likely among economic migrants, a result of disparities in relative economic attractiveness between districts. The concentration of mobility around towns is logical as they are economic and demographic epicentres in the study regions. Moreover, development corridors or commercially linked areas could also show high human mobility. In Abobo district of Gambella the observed human mobility was considerably high and this could be attributed to large-scale commercial agriculture and gold mining that has increased the economic significance of the area in recent years. Abobo town, which is close to these development activities, was the centre of mobility observed in the district (Figs. 3, 5).

Hourly human mobility patterns differed starkly between the two study regions, highlighting the importance of contextual knowledge for public health efforts. Two peaks (one in the morning and the other one in the evening) were observed in both Benishangul-Gumuz and Gambella (Figs. 6, 7). One of the largest defenses against malaria spreading mosquitoes in these regions are bed nets, which are most commonly used inside homes and at nighttime. Presumably, geographic movements that are large enough to trigger a mobile phone handover indicate times whereby individuals are not under a bed net in their respective homes. While there is a dearth of published research on the entomology of malaria in both regions, a recent study in Benishangul-Gumuz by Dugassa et al. [23] reported that the peak *Anopheles* biting time observed around agricultural development areas is from 7 pm to10pm, 12am to 2am, and 3 to 5am (Fig. 11). Likewise, a recent study by the authors in Gambella indicated that outdoor *Anopheles* feeding behaviour experienced peaks from 7 to 8 pm, 10 to 11 pm, 2 to 3am, and 4 to 5am. Indoor feeding increased throughout much of the early nighttime hours, with less clear peaks (Fig. 12). Importantly, there was relatively high feeding behaviour outside of these peak hours in both study sites (for example, from 6 to 7 pm and from 5 to 6am). A comparison of human movements (that would be occurring outside of the home) and Anopheles feeding behaviour suggest that bed nets are unlikely to be sufficient to disrupt human-mosquito vector contact in these regions [36]. This may be important to consider for regional malaria elimination goals.

Likewise, there were significant in and out movements of people in and from these two regions. The high movement of people into these regions during peak transmission seasons (September to December) could increase the pool of susceptible individuals in these malarious settings. Many migrants are from areas of low or no malaria transmission (highland population centres) and are therefore immunologically naïve, potentially experiencing more severe symptoms upon infection [37]. Likewise, movements out of the region during peak transmission season may contribute to spread or reintroduction of parasites to regions that are susceptible to malaria transmission but have achieved control or local elimination. Movements of infectious individuals could, therefore, reverse successes in malaria control and hence challenge the national initiative to embark on malaria elimination.

## Conclusions

This study revealed empirical human mobility and travel activities in hourly, weekly, and monthly bases in two regions of heightened importance for malaria epidemiology in the Horn of Africa. This research shows significant hourly movements during times when important mosquito vectors are active, which has relevance for vector-focused interventions, such as bed nets. Likewise, this research shows significant in and out-movements of individuals to and from these regions during times of heightened malaria transmission. This may impede the national effort to eliminate malaria from Ethiopia. Finally, this study illustrates the usefulness of cellular network data for empirically measuring human movements in this region. Due to the diversity of people’s daily movements, modelling asynchronously time-varying human mobility pattern may be worth further research both in these regions and beyond, with implications for malaria control and elimination, as well as for other diseases of importance.

## Supplementary Information

**Additional file 1:** Human mobility model and analysis detail.

## Data Availability

Data supporting the result are included within the article.

## Abbreviations

UMTS: Universal mobile telecommunication system
GSM: Global system for mobile communications
LTE: Long term evolution
BBC: Break-before-connect
UE: User equipment
CBB: Connect (Entry)-before-break
HO: Handover
RRC: Radio resource control
e NB: Evolved Node B
3GPP: 3Rd generation partnership project
BS: Base station
MR: Measurement report
UL: Upper link
DL: Down link
SINR: Signal-to-interference-plus-noise ratio
OSS: Operation support system
TCH: Traffic channel
SDCCH: Standalone dedicated control channel
