# Supplementary material for: Mobile phone handover data for measuring and analysing human population mobility in Western Ethiopia: implication for malaria disease epidemiology and elimination efforts
Source: Malar J. 2022 Nov 11;21:323. doi: 10.1186/s12936-022-04337-w (PMC9652832; doi:10.1186/s12936-022-04337-w)
Supplement: Supplementary file 1 — Additional file 1: Human mobility model and analysis detail. [file 12936_2022_4337_MOESM1_ESM.docx]

**SUPPLEMENTARY MATERIALS**

**Basic concepts of the proposed approach**

Handover (HO) refers to the transfer of a user’s (UE) connection from one radio channel to another channel (can be the same or different cell). Handoffs are broadly classified into two categories—hard and soft handoffs which are also known as Break-Before-Connect (BBC) and Connect (Entry)-Before-Break (CBB), respectively. The hard handoff can be further divided into two different types— intra- and inter cell handoffs. The soft handoff can also be divided into two different types—multi way soft handoffs and softer handoffs [31, 32, 33, 34].

The handover process is performed mainly via the radio resource control (RRC) layer between UE, serving eNB (evolved node B) and Target eNB in the control-plane as per the 3GPP LTE standard and has been described by Perez and his colleagues [35]. eNB is the hardware that is connected to the mobile phone network that communicates directly and wirelessly with UEs. The HO is triggered by the eNB, based on the received measurement reports from the UE and the decisions are based on the signal strength measurements of the neighboring BSs done at the UE. However, due to the user’s mobility, the conditions or decision of a handover algorithm could vary over time.

Generally, the handover procedure consists of three phases: preparation, execution, and completion phases. In addition to this a HO process completes into five different key steps. One: The UE measures the downlink signal strength periodically. Two: The UE processes the measurement results. Three: The UE sends the measurement report (MR) to the serving eNB based on predefined HO criteria. Four: After receiving the MR from UE, the Serving eNB takes the handover decision based on the received MR and issues a handover request message to the target eNB. Five: The UE receives a handover command from the serving eNB and data forwarding from serving eNB to target eNB starts and the serving eNB sends a handover command (it completes the handover) [34].

**Approach for quantification of human mobility**

For this study we considered the geographical displacements $r=\left| X_{2}-X_{1} \right|$ between a first $(x_{1})$ and secondary $(x_{2})$ measurement location with the elapsed time t between successive reports. For dense urban, urban and rural it becomes $\left| X_{2}-X_{1} \right|$ $\leq300m$, $\left| X_{2}-X_{1} \right|$ $\leq3Km$ and $\left| X_{2}-X_{1} \right|$ $\leq5 km$ respectively. These different spatial extents were used because mobile phone tower distribution is spatially clustered in urban settings and much more dispersed in rural settings.

The average handovers per UE per unit of time represents the number of handovers that occurs during a time under consideration. With the total number of successful handovers${HO}_{Total}$, and the total number of users$J$, the average handovers per UE per second ${HO}_{avg}$ can be defined [36].

$${HO}_{avg}=\frac{{HO}_{Total}}{JxT}$$

Where, *J* and *T* are the total number of users and total time under consideration, respectively.

Using mobile phone users, Gonzalez et al.(2008) showed that the distribution of displacements over all users is well approximated by a truncated power-law with an exponential cut-off $\left( P\left( \triangle r \right)=\left( \triangle r+\triangle r_{o} \right)^{-\beta}\exp\left( -\frac{\Delta r}{k} \right) \right)$ , $where, \triangle r$ =frequently called jump size, $\beta$ =$1.75\pm0.15 ,$(mean $\pm$standard deviation), $\Delta r_{o}=1.5 km, and k a cut-off value$varying in different experiments) and showed that individual truncated Levy trajectories co-exist with population-based heterogeneity. This heterogeneity was measured in terms of the radius of gyration distribution$P(r_{g})$, by calculating $r_{g}$ for distance traveled by a user. Meaning, most of people usually travel in close vicinity to their location, while few frequently make long journeys [16].

### For this study, in the context of individual-mobility the probability that an individual moves to a new geographical location ( distance $r$ in a time $\triangle t$) is proportional to the modified handover $f(r)$ value , $HO\times\delta$, where $\delta$ is the total number of a scaling multiplicative factor that considers the probability of Handover distribution (UL/DL quality, UL/DL interference, UL/DL level, power budget, SINR, near moving mobile station, etc) in different geographical location and time/ season (hr., day, month and year). With this approach we also get the total number of discrete moves the active individual mobile user had up to time t.

By considering the individual mobile communications at a site level, we obtain a network of 20 districts in Beneshangule and 13 districts in Gambella. We deﬁned the intensity of mobility between areas (locations) A and B as

$$M_{AB}=\sum_{i\in A,j\in B} m_{ij .}$$

We also defined the probability of human mobility between the areas (locations) A and B by considering ﬂowing within a location, into location and out of location scales with active mobile user size, respectively as follows

$$M_{AA}=\sum_{i\in A,j\in A} m_{ij .}$$

$${M*}_{A}=\sum_{i\notin A,j\in A} m_{ij .}$$

$$M_{A*}=\sum_{i\in A,j\notin A} m_{ij .}$$

Given the inputs, the algorithm computes for every pair of locations i, j the probability of moving from i to j (Algorithm 1,).

The probability that a call goes through at least $N$ handovers is specified in [37].

$$P_{r}\left[ handovers \geq N \right]=\int_{0}^{\infty} P_{r}\left[ T_{N}\leq t \right]pdf_{c}\left( t \right)dt$$

Where $P_{r}\left[ T_{N}\leq t \right]$ is the probability that the $N\_th$ cell boundary crossing will take place before time t, and $pdf_{c}\left( t \right)$ is the call duration probability density function.

**Algorithm**

For this study, when creating a representative and realistic version of quantifying human mobility, several steps have to be taken. The process starts with initiating the cell location and deciding on which fundamental characteristics the human mobility quantification approach should consist including mobility inputs, set threshold for short distance movement and compute which observations are needed.

The following algorithm was used for generating mobility model using cellular network data from Benishangul-Gumuz and Gambella regions. First the necessary cell towers in both 2G and 3G sites were selected (the cell towers where a person makes consecutive calls). For each cell the necessary mobility parameter data were collected per hour, week and month. Second, the scale factor was computed to change the mobility parameter value to the human mobility pattern. Finally, the relative human mobility probability for each scenario was calculated. Generally, several preprocessing steps had been carried out before analysis.
